# Clinical Manifestations of Aortocaval Fistulas in Ruptured Abdominal Aortic Aneurysm: Report of Two Cases

**DOI:** 10.1155/2012/123081

**Published:** 2012-10-24

**Authors:** Emmanouil D. Psathas, Stella Lioudaki, Mikes Doulaptsis, Petros Charalampoudis, Chris Klonaris, Chris Verikokos

**Affiliations:** Second Department of Propaedeutic Surgery, Laiko Hospital, Medical School, University of Athens, 11527 Athens, Greece

## Abstract

Aortocaval fistula (ACF) is an unusual complication of ruptured abdominal aortic aneurysm (AAA), involving less than 3–6% of all ruptured cases. The clinical presentation is often obscure, depending on the coexistence of retroperitoneal rupture and hemodynamic instability. Prompt preoperative diagnosis is essential in order to plan the operative approach and improve patient's outcome. We report the surgical treatment of two patients presented in the emergency department with ACF due to ruptured AAA, each with different clinical presentation, emphasizing the high index of suspicion needed by the clinician to early diagnose and treat this often lethal condition. Operative strategy and special considerations in the management of this subgroup of patients are also discussed.

## 1. Introduction

Rupture of an abdominal aortic aneurysm into the inferior vena cava (IVC) is an uncommon and often devastating condition, involving less than 3–6% of all ruptured aortic aneurysms [1, 2]. Aortocaval fistula can be combined with or without retroperitoneal rupture, in which case the clinical presentation can be obscure, mimicking other cardiovascular conditions that cause lower extremities' edema and congestive heart failure. Therefore, a high index of suspicion is vital for the clinician to early diagnose and treat this often lethal surgical emergency. We report two cases of ruptured AAA with concomitant aortocaval fistulation, treated with conventional open repair.

## 2. Case Reports

### 2.1. Case 1

**Figure 1:**
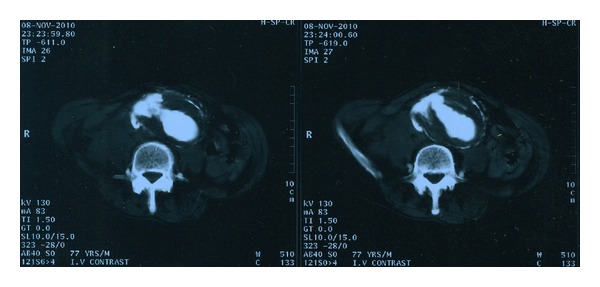
Abdominal CT identifying a large 7.8 cm AAA with evidence of retroperitoneal rupture. Notice the synchronous contrast filling of the aorta and IVC and their communication through the sac, indicating aortocaval fistulation.

**Figure 2:**
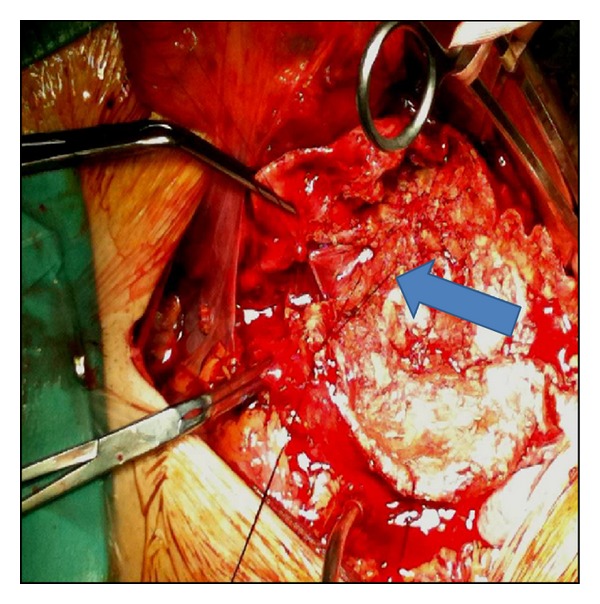
The aortocaval fistula was oversawn from within the sac. Finally, hemostasis was obtained by infrarenal IVC and iliac veins ligation.

A 77-year-old male patient was admitted as an emergency after a collapse. On admission, he was pale and anxious; his blood pressure was 80/40 mmHg with a heart rate of 120/min. He complained of lumbar and lower extremities pain for the past 6 hours. Physical examination revealed a pulsative abdominal mass with a profound thrill in the lower right abdominal wall. A loud systolic bruit was heard during abdominal auscultation—especially to the right, and there was an obvious jugular venous engorgement, with normal sinus tachycardia on ECG examination. The diagnosis of a ruptured AAA with aortocaval fistula was confirmed by emergency CTA scan. This revealed a 7.8 cm infrarenal AAA extending distally to the left common iliac artery, retroperitoneal hematoma, and simultaneous contrast filling of the abdominal aorta and the inferior vena cava (Figure 1). The patient was transferred urgently to the operating room for planned open repair of his aneurysm and aortocaval fistula. After midline incision, the infrarenal aorta and iliac arteries were controlled and cross-clamped. Following opening of the aortic sac, a large amount of venous back bleeding appeared from within the sac, and temporary control was accomplished with digital compression. After removal of the intramural thrombus, a 30 × 20 mm fistula was identified and oversewn from within the sac of the aneurysm. The bleeding was only partially controlled and ligation of the infrarenal IVC and iliac veins was deemed necessary in order to obtain hemostasis. Finally, a bifurcated 18–9 mm Dacron graft was placed to restore the blood flow to the lower extremities (Figure 2). After a 6-day stay in the intensive care unit (ICU), the patient was discharged on the 14th postoperative day, with no signs of peripheral oedema or DVT. Followup duplex scan examination three months later confirmed the interruption of the infrarenal IVC, with extensive collateral vein network via the hemiazygous and the pancreaticoduodenal vein. Both internal and external iliac veins were patent bilaterally, and there where no signs of vein thrombosis or pelvic congestion syndrome. The patient remains well after 18 months.

### 2.2. Case 2

**Figure 3:**
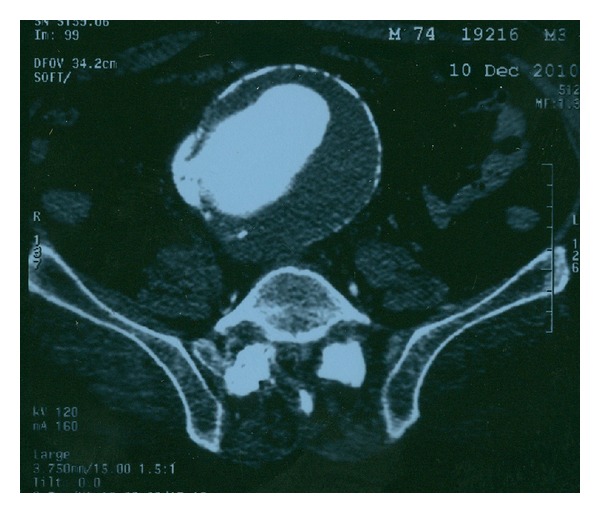
Abdominal CTA revealing a large 9.8 cm AAA with communication between the aorta and the IVC, without evidence of retroperitoneal rupture.

A 78-year-old male patient with a history of arterial hypertension, chronic renal failure (Creatinine 2.4 mg/dL), smoking, and morbid obesity was admitted to the emergency department due to exertional dyspnea, shortness of breath, and recurrent episodes of acute pulmonary edema, without any obvious cardiological reason. At presentation, he was haemodynamically stable (heart rate 100/min, BP 120/85 mmHg). Physical examination revealed bilateral pulmonary rales, lower extremities edema, and—despite obesity—a large pulsative mass was noticed during abdominal examination. There were no bruits or thrills noticed during initial examination. Emergency CTA scan revealed a large 9.8 cm infrarenal AAA with communication between the aorta and the IVC and no signs of retroperitoneal rupture (Figure 3). Upon emergency laparotomy and aortic cross-clamping, a 20 × 15 mm aortocaval communication was found after opening of the aortic sac. Digital compression was utilized to control the bleeding from the IVC, and the fistula was oversawn from within the sac with monofilament sutures. A 20 mm Dacron tube graft was placed to restore perfusion to the lower extremities. Postoperatively, the patient was transferred to the ICU, where he was put on continuous venovenous haemofiltration due to acute renal failure, which resolved on POD 4. The patient was extubated on the 10th postoperative day and was discharged after 25 days of hospitalization, with bilateral peripheral edema but no evidence of DVT. His edemas' have resolved after 3 months, and the patient remains well 16 months after operation.

## 3. Discussion

Over 80% of reported aortocaval fistulas are related with ruptured abdominal aortic aneurysms. Other causes refer to penetrating trauma, mycotic aneurysms, Takayasu's arteritis, and connective tissue diseases [3]. Rupture into the vena cava may be asymptomatic and recognized during elective AAA repair or may be overlooked when symptoms of rupture predominate. However, preoperative diagnosis is essential, in order to minimize blood loss and avoid possible intraoperative pulmonary embolism [4, 5].

A typical clinical presentation includes sudden onset of abdominal pain, shortness of breath, and a pulsative abdominal mass with an audible machinery-like bruit and/or a thrill [6]. Nevertheless, symptoms seem to be related with the hemodynamics of the communication. In large, high-flow aortocaval fistulas, symptoms of cardiac failure, and sudden central venous hypertension with no clear cause may be the only findings suggesting the diagnosis [7]. Intracaval rupture of an abdominal aneurysm causes a sudden fall of peripheral vascular resistance with concomitant increase of venous pressure. This leads to an increase of cardiac rhythm and stroke volume (SV), which results in myocardial hypertrophy, sinus dilatation, and finally cardiac failure.

Contrast CT in patients with suspected aortocaval communication is diagnostic in the majority of cases, as long as it is allowed by the patients' hemodynamic status. Pathognomonic findings include indentation and fistula line in the vena cava, disappearance of the fatty planes between vena cava and aorta, and rapid simultaneous contrast passage into the vena cava from the aorta [8].

Patients with aortocaval communication should be operated immediately. Mobilization of the aneurysm should be made as gentle as possible, in order to avoid paradox pulmonary embolism from dislodgment of debris from the aneurysm sac to the vena cava. Aortic vascular control should be obtained first, while a few techniques have been described to control the bleeding from the vena cava. Most frequently, digital or sponge compression of the IVC is obtained from within the sac, while other techniques, like insertion of occluding balloon catheters, have been described to avoid massive hemorrhage or air embolism. Closure of the fistula should be done from within the aneurysm sac, using monofilament mattress sutures. In cases that this is not possible, ligation of the infrarenal IVC and/or iliac veins can be applied in order to obtain hemostasis [9]. Complications expected after IVC ligation include leg edema (30%), recurrent DVT (16%), venous pelvic compression syndrome, and venous claudication, although it is well tolerated in most cases [10].

A few reports have been published over the past years regarding successful endovascular treatment of aortocaval fistulation. Although most of them report favorable results concerning early survival and short hospitalization, persistent type II endoleak is a matter of concern, while long-term followup is missing [11–15].

## 4. Conclusion

Aortocaval fistula is an uncommon complication of large AAAs and can occur with or without retroperitoneal rupture, in which case, signs of congestive heart failure might predominate. This fact stretches the importance of thorough abdominal examination—including auscultation—by any cardiologist evaluating a patient with sudden-onset right heart failure and no obvious cardiac reason. A high index of clinical suspicion along with prompt diagnosis and surgical treatment is of outmost importance in the management of this devastating condition. Although endovascular techniques have reported good early results, open surgery with special considerations regarding intraoperative maneuvers remains the cornerstone of treatment.
